# Editorial for the Special Issue on SiC Based Miniaturized Devices

**DOI:** 10.3390/mi11040405

**Published:** 2020-04-13

**Authors:** Stephen E. Saddow, Daniel Alquier, Jing Wang, Francesco LaVia, Mariana Fraga

**Affiliations:** 1 Department of Electrical Engineering, University of South Florida, 4202 East Fowler Avenue, ENB118 Tampa, FL 33620, USA; 2 GREMAN UMR-CNRS 7347, Université de Tours, 16 rue Pierre et Marie CURIE, BP 7155, 37071 Tours Cedex 2, France; 3 CNR-IMM Sezione di Catania, Strada VIII 5-Zona Industriale, I-95121 Catania, Italy; 4 Instituto de Ciência e Tecnologia, Universidade Federal de São Paulo (UNIFESP), Rua Talim 330, São José dos Campos 12231-280, Brazil

The MEMS devices are found in many of today’s electronic devices and systems, from air-bag sensors in cars to smart phones, embedded systems, etc. Increasingly, the reduction in dimensions has led to nanometer-scale devices, called NEMS (Nano-Electrical-Mechanical Systems). The plethora of applications on the commercial market speaks for itself, and especially for the highly precise manufacturing of silicon-based MEMS and NEMS. While this is a tremendous achievement, silicon (Si) as a material has some drawbacks, mainly in the area of mechanical fatigue and thermal properties. Silicon carbide (SiC) is a well known wide-bandgap semiconductor whose adoption in commercial products is experiencing exponential growth, especially in the power electronics arena. While SiC MEMS have been around for decades, in this Special Issue we sought to capture both an overview of the devices that have been demonstrated to date, as well as bring new technologies and progress in the MEMS processing area to the forefront. This Special Issue contains one review paper and nine original research papers, with both experimental and theoretical investigations, reporting the recent progress of SiC materials, processing, modeling and device technology.

The review paper of this Special Issue provides an overview of high-temperature SiC power electronics, with a focus on high-temperature converters and MEMS devices [1]. This paper mainly surveyed the research and development of SiC-based high-temperature converters as well as the existing technical challenges facing high-temperature power electronics, including gate drives, current measurements, parameters matching between each component and packaging technology.

The discussion on the original research published in this Special Issue opens with the paper on the development of a 1200V/200A full-SiC half-bridge power module by Zhang et al [2]. Their study focused on the influences of output power on the turn-on Vgs characteristics for high-power and high-frequency application. There is also a paper addressing the design and simulation of an improved 4H-SiC metal semiconductor field effect transistor (MESFET) based on the double-recessed MESFET (DR-MESFET) [3].

The use of SiC in radiation detection is the subject of two papers in this issue. Mandal et al. investigated the development of miniature 4H-SiC-based radiation detectors for harsh environment application [4], whereas Puglisi et al. reported the electrical and spectroscopic performance of an innovative position-sensitive semiconductor radiation detector in epitaxial 4H-SiC [5].

The mechanical properties of hexagonal SiC (4H- and 6H-SiC) are also discussed in this Special Issue. Ben Messaoud et al. reported the Young’s modulus and the residual stress of 4H-SiC circular membranes on 4H-SiC substrates [6]. Pan et al. approached the mechanical behavior and material-removal mechanisms of single crystal 6H-SiC under the effects of abrasives by combining the morphologies of the machined surfaces and the results of nanoindentation experiments are described [7]. Chai et al. proposed a theoretical model of the critical depth of cut of nanoscratching on a 4H-SiC single crystal with a Berkovich indenter [8].

The last two articles of this Special Issue involve the synthesis, characterization and application of SiC films. Galvão et al. explored the structural and chemical properties of polycrystalline SiC films grown at room temperature on Si and aluminum nitride (AlN)/Si substrates by the high-power impulse magnetron sputtering (HiPIMS) technique [9]. Beygi et al. reported on the design and fabrication of a Michigan-style SiC neural probe on a silicon-on-insulator (SOI) wafer for the ease of the manufacture. The probe is composed of 3C-SiC, which was epitaxially grown on a SOI wafer [10]. These neural interfaces may pave the way for long-term human implants to treat such serious conditions as Parkinson’s, dementia and depression, restore lost functionality due to brain damage and enable seamless integration of robotic prosthetics for patients who have suffered limb-loss.

We sincerely hope that this Special Issue on SiC-based miniaturized devices can be a valuable source of information for researchers working on this topic. We would like to thank all the authors and reviewers for their contribution and effort. We are also grateful to the editorial and production staff of Micromachines for their support. We hope that you enjoy reading this Special Issue.
